# TNFR2 expression predicts the responses to immune checkpoint inhibitor treatments

**DOI:** 10.3389/fimmu.2023.1097090

**Published:** 2023-02-14

**Authors:** Ping Liao, Mengmeng Jiang, Md Sahidul Islam, Yiru Wang, Xin Chen

**Affiliations:** ^1^ Institute of Chinese Medical Sciences, State Key Laboratory of Quality Research in Chinese Medicine, University of Macau, Macao, Macao SAR, China; ^2^ Department of Pharmceutical Sciences, Faculty of Health Sciences, University of Macau, Macao, Macao SAR, China; ^3^ MoE Frontiers Science Center for Precision Oncology, University of Macau, Macao, Macao SAR, China; ^4^ Guangdong-Hong Kong-Macau Joint Lab on Chinese Medicine and Immune Disease Research, Macao, Macao SAR, China

**Keywords:** TNFR2, immune checkpoint inhibitors, tumor microenvironment, CD4 + Foxp3 + regulatory T cells, exhausted CD8 T cells, biomarker

## Abstract

Immune checkpoint inhibitors (ICIs) by targeting PD-1/PD-L1 or CTLA-4 have markedly improved the outcome of cancer patients. However, most solid tumor patients can’t benefit from such therapy. Identification of novel biomarkers to predict the responses of ICIs is crucial to enhance their therapeutic efficacy. TNFR2 is highly expressed by the maximally immunosuppressive subset of CD4^+^Foxp3^+^ regulatory T cells (Tregs), especially those present in tumor microenvironment (TME). Since Tregs represent a major cellular mechanism in tumor immune evasion, TNFR2 may be a useful biomarker to predict the responses to ICIs therapy. This notion is supported by our analysis of the computational tumor immune dysfunction and exclusion (TIDE) framework from published single-cell RNA-seq data of pan-cancer databases. The results show that, as expected, TNFR2 is highly expressed by tumor-infiltrating Tregs. Interestingly, TNFR2 is also expressed by the exhausted CD8 T cells in breast cancer (BRCA), hepatocellular carcinoma (HCC), lung squamous cell carcinoma (LUSC), and melanoma (MELA). Importantly, high expression of TNFR2 is associated with poor responses to the treatment with ICIs in BRCA, HCC, LUSC, and MELA. In conclusion, the expression of TNFR2 in TME may be a reliable biomarker for the precision of ICIs treatment of cancer patients, and this idea merits further research.

## Introduction

The development of immune checkpoint inhibitors (ICIs) by targeting CTLA-4, PD-1 or PD-L1 has now revolutionized cancer treatment (1–3). ICIs interrupt co-inhibitory signaling pathways and relieve or overcome tumor-induced immunosuppression, thereby reinvigorating anti-tumor immune responses (2, 4). Despite the outcomes being promising, the majority of patients cannot derive the benefits from PD-1/PD-L1 and CTLA-4 inhibitors therapy, due to low response rates and immune-related adverse events (irAEs) (5–8). The onset of irAEs is highly unpredictable, severe, and sometimes even fatal. The identification of novel biomarkers to predict the responses of ICIs is crucial to enhance the efficacy of their treatments. Recently, a substantial number of studies have been performed to explore the potential irAE biomarkers, including cytokines/chemokines, autoantibodies, immunogenetics, and microbial biomarkers (9–13). To date, no single biomarker has been identified to precisely predict the responses in patients receiving ICIs treatment.

CD4^+^Foxp3^+^ regulatory T cells (Tregs) are central regulators of anti-tumor immune responses (14) and they represent a major cellular mechanism of tumor immune evasion (15, 16). Tumor necrosis factor receptor 2 (TNFR2), one of two receptors that mediate the biological function of TNF, is predominantly expressed by highly immunosuppressive subset of Tregs in mice and humans (17–20). There is now compelling evidence indicate that TNFR2 plays a decisive role in the activation, proliferative expansion, immunosuppressive function, and phenotypic stability of Tregs (17, 19, 21). Furthermore, TNFR2 is also expressed by other types of immunosuppressive cells such as myeloid-derived suppressor cells (MDSCs), mesenchymal stem cells (MSCs), and some tumor cells (16, 22, 23). We have proposed that TNFR2 behaves as an immune checkpoint stimulator and oncoprotein (15). Interestingly, it was reported that TNF could upregulate PD-L1 expression in pancreatic cancer cells through TNFR2 signaling, and consequently induced PD-1/PD-L1-mediated immune evasion (24). Targeting TNFR2 was able to significantly improve the anti-tumor efficacy in multiple tumor models (25–27). We further hypothesize that TNFR2 may be a useful biomarker for the prediction of the responses to ICIs therapy.

## TNFR2 is preferentially expressed by tumor-infiltrating Tregs and exhausted CD8 T cells

**Figure 1:**
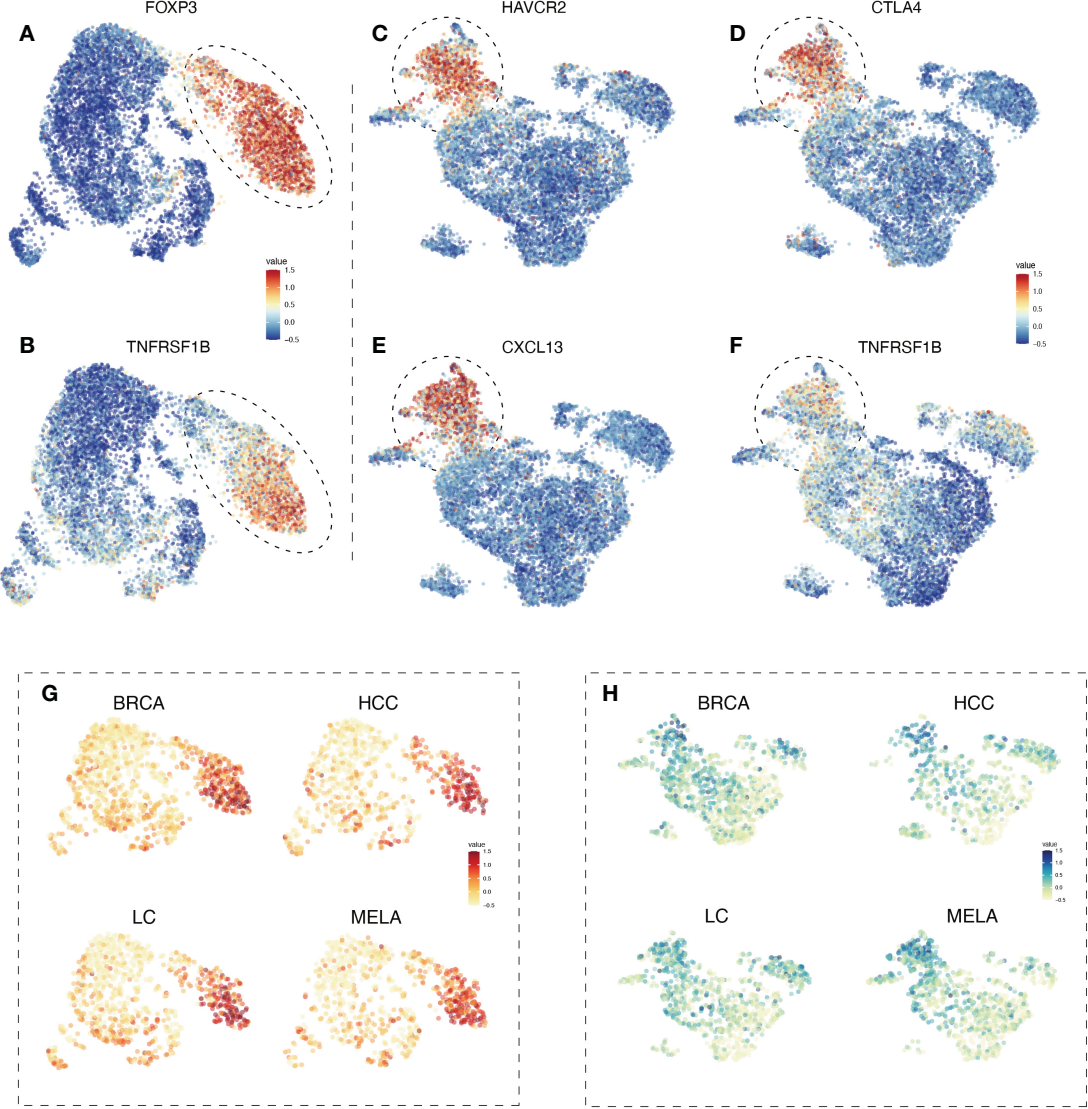
The expression profile of TNFR2 in tumor-infiltrating Tregs and exhausted CD8 T cells in BRCA, HCC, LC, and MELA. To estimate the differentiation of TNFR2 expression among tumor-infiltrating immune cells, we re-analyzed and integrated the pan-cancer single-cell landscape from the Zemin Zhang group’s study, based on their framework of scDVA (short for single-cell RNA-seq data visualization and analysis). UMAP visualization shows the expression of FOXP3 **(A)** in CD4 T cell metaclusters. The dotted line represents regulatory T cells. **(B)** TNFR2 expression in CD4 T cell metaclusters. In CD8 T cell metaclusters, the expression of selected marker genes, such as HAVCR2 **(C)**, CTLA4 **(D)**, and CXCL13 **(E)** are shown. Dotted line represents terminal exhausted CD8 T cells. **(F)** TNFR2 expression in CD8 T cell metaclusters. UMAP visualization shows the expression of TNFR2 in CD4 T cell metaclusters **(G)** and CD8 T cell metaclusters **(H)** across four cancer types (BRCA, HCC, LC, and MELA).

To test the hypothesis, we first examined the expression of TNFR2 in different types of tumor-infiltrating T cells, by re-analyzing and integrating the pan-cancer single-cell landscape with UMAP visualization from the Zemin Zhang group’s study (28), based on their framework of scDVA (short for single-cell RNA-seq data visualization and analysis) among those various tumor types. It was reported by us and others that TNFR2 could be expressed by mouse antigen-experienced CD4^+^Foxp3^-^ conventional T cells, including those in the tumor environment (29, 30), we thus firstly analyzed TNFR2 expression by subsets of cells in CD4 T cell metaclusters. As expected, the expression of TNFR2 by CD4^+^FoxP3^+^ Tregs was much higher than that expressed by conventional CD4 T cells in human cancers (**Figures 1A, B**). Therefore, high expression of TNFR2 remained to be a trustful marker of Tregs in human tumors.

CD8 T cells could also express TNFR2 and the signal of TNFR2 in CD8 cytotoxic T lymphocytes (CTLs) was purportedly mediated by the anti-tumor effect of agonistic antibodies against TNFR2 (27). We analyzed TNFR2 expression on tumor-infiltrating CD8 T cells in human cancers. The results showed that CD8 T cells indeed expressed high levels of TNFR2. Interestingly, CD8 cells with high TNFR2 expression appeared to have a terminal exhausted phenotype, as defined by the expression of CTLA4, HAVCR2, and CXCL13 (**Figures 1C–F**). Furthermore, we analyzed the expression of selected marker genes across several cancer types. UMAP visualization demonstrated that high expression of TNFR2 was presented in tumor-infiltrating Tregs and exhausted CD8 T cells across human breast cancer (BRCA), hepatocellular carcinoma (HCC), lung cancer (LC), and melanoma (MELA) (**Figures 1G, H**). TNFR2 expression by CD4 T and CD8 T cell metaclusters in other 17 human cancer types were analyzed as well, and results consistently showed that TNFR2-expressing cells are mostly concentrated by Tregs in CD4 T cells, while distribution of TNFR2-expressing cells are less homogenous in the CD8 T cells in these cancer types (**Supplementary Figure 1**). Besides, the expression profile of TNF in tumor-infiltrating T cells was also analyzed. The results showed that there was no clear pattern of TNF expression in CD4 and CD8 T cells (**Supplementary Figure 2**).

## Association of TNFR2 with responsiveness to ICIs therapy

**Figure 2:**
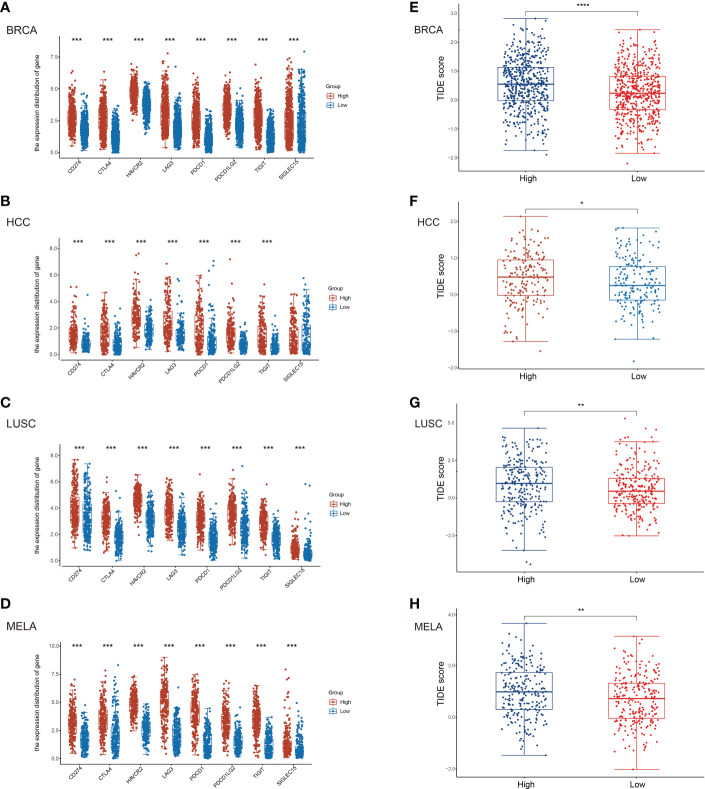
Correlation of TNFR2 expression and immune checkpoint gene markers. To predict the correlation between TNFR2 expression and the responses to immune checkpoint inhibitor therapy, (TIDE) score was evaluated. According to the median value of TNFR2 expression, tabulated tumor samples and types (BRCA, HCC, LUSC, and MELA) were divided into TNFR2^high^ and TNFR2^low^ groups. Then, TIDE score was used to predict the correlation between TNFR2 expression and immune checkpoint inhibitor responses. The correlation between TNFR2 and representative immune checkpoints gene markers, such as CD274 (PD-L1), CTLA4, HAVCR2, LAG3, PDCD1 (PD-1), PDCD1LG2 (PD-L2), TIGIT, and SIGLEC15 were shown in BRCA **(A)**, HCC **(B)**, LUSC **(C)** and MELA **(D)**. TIDE score was used to predict the correlation between TNFR2 expression and ICIs responses in BRCA **(E)**, HCC **(F)**, LUSC **(G)**, and MELA **(H)**. *P < 0.05, **P < 0.01, ***P < 0.001, ****P < 0.0001.

To further assess the potential relationship of TNFR2 signaling on the state of the immune-suppressive tumor environment, and to predict the response of patients to the treatment with ICIs, we evaluated the expression of immune-checkpoint–relevant gene markers of SIGLEC15, TIGIT, CD274 (PD-L1), HAVCR2, PDCD1 (PD-1), CTLA4, LAG3, and PDCD1LG2 (PD-L2) (31, 32). The results showed that TNFR2 expression was positively correlated with the expression of these checkpoint markers in BRCA, HCC, LUSC, and MELA, respectively (**Figures 2A–D**). Next, we assessed the correlation between TNFR2 expression and the ICIs response by Tumor Immune Dysfunction and Exclusion (TIDE) score (33). The results showed that higher TNFR2 expression was associated with higher TIDE scores, complying with poorer responses to ICIs therapy, in patients with BRCA, HCC, LUSC, and MELA (**Figures 2E–H**). Therefore, the results of our analysis clearly indicate that TNFR2 expression is associated with a general immunosuppressive state in tumor environments. Thus, high levels of TNFR2 expression are associated with poor responses of patients to ICIs treatment.

## Discussion and perspective

We and others have shown that targeting TNFR2 with antagonistic antibodies could mobilize anti-tumor immune responses by eliminating Treg activity, yielding a potent anti-tumor effect both in mice and humans (25–27, 34–36). Fcγ-binding could result in the conversion of antagonistic TNFRSF receptor-specific antibodies into strong agonists (37), therefore, further investigation is needed to clarity if the anti-tumor effect of these antibodies is truly caused by the inhibition of the TNFR2 signal. In fact, it was shown that TNFR2 agonistic antibody Y9 could inhibit tumor growth by acting directly on CD8 CTLs in tumors and stimulating their activation and expansion in murine cancer models (27). Based on the above contradictory results, pharmacological companies are currently developing both agonistic and antagonistic anti-TNFR2 antibodies simultaneously (38), in chance one of them may eventually be proved useful in the clinic as a cancer immunotherapeutic agent. Therefore, further understanding TNFR2 expression by T cell subsets in tumor microenvironment is important to clarify the primary cellular target of both agonistic and antagonistic TNFR2 antibodies. In this study, we found that among tumor-infiltrating CD4 T cells, TNFR2 is quite selectively expressed by Foxp3^+^ Tregs. Notably, although Foxp3 expression is a standard marker for murine Tregs, caution should be taken to use Foxp3 as a marker for human Tregs, since it can also be expressed by the activated human T cells upon TCR triggering (39). Thus, further study is needed to verify if all Foxp3-expressing cells in the metacluster are bona-fide Tregs or not. The expression of TNFR2 by tumor-infiltrating CD8 T cells appeared to be less homogeneous and relatively discrete, as compared with its expression in CD4 T cells. Nevertheless, it is clear that TNFR2 is mainly expressed by CD8 T cells with an exhaustive phenotype. Our results are consistent with a number of previous studies that suggest both Tregs and exhausted CD8 CTLs are potential targets of TNFR2 antibody treatments (25, 27, 34, 35, 40). Based on our results, it is possible to propose that antagonistic antibodies may dampen the immunosuppressive function of Tregs, while the exhaustive phenotype of CD8 CTLs may be reversed simultaneously.

Our results also indicate that TNFR2 expression levels are associated with the responsiveness of ICIs treatment. The expression of TNF, an endogenous ligand of TNFR2 with the capacity to up-regulate TNFR2 expression by Tregs (41), in tumor tissue could be markedly upregulated after ICIs treatment (42). It was shown that TNF derived from CD8^+^ T cells and CD8^-^ T cells represent a crucial effector mechanism in ICI-responsive tumors (43). Nevertheless, it was also reported that blockage of TNF overcome the resistance to anti-PD-1 treatment in experimental melanoma (44), and the treatment with TNF inhibitors enhanced the anti-tumor effect of combined CTLA-4 and PD-1 immunotherapy (45). However, a preclinical study revealed that the treatment with anti-mouse TNFR2 surrogate antibody (suAb) abrogated TNF-induced expansion of Tregs *in vitro* and decreased expression of PD-1 on CD8 tumor-infiltrating lymphocytes (TILs) *in vivo* (46). Thus, it is highly possible that TNF-TNFR2 axis is attributable to the resistance to ICIs therapy. This notion is supported by the evidence from us and others that blockade of TNFR2 with antagonistic antibodies could markedly enhance the efficacy of ICIs treatment in mouse tumor models (25, 35, 47). For example, in a cohort of metastatic melanoma patients treated with CTLA4 and PD-1 blockade, a deep immune analysis of tumor samples found that TNF was markedly upregulated after PD-1 treatment (42).

Taken together, our analysis clearly indicates that the expression of TNFR2 in TME may be a reliable biomarker for the precision ICIs treatment of cancer patients. Our results also support the notion that both tumor-infiltrating Tregs and exhausted CD8 CTLs are major cellular targets of antagonistic TNFR2 antibody treatment, for the reason that both subsets of tumor-infiltrating lymphocytes express high levels of TNFR2. These possibilities merit further clinical research.

## Data availability statement

The original contributions presented in the study are included in the article/**Supplementary Material**. Further inquiries can be directed to the corresponding author.

## Author contributions

PL and MJ designed the study, PL, MJ, YW and MI analyzed and interpreted the data, and drafted the manuscript. XC, the corresponding author, was involved in designing the work and approving the final version to be published. All authors agree to submit the manuscript.
