# Supplementary material for: TNFR2 expression predicts the responses to immune checkpoint inhibitor treatments
Source: Front Immunol. 2023 Feb 14;14:1097090. doi: 10.3389/fimmu.2023.1097090 (PMC9971721; doi:10.3389/fimmu.2023.1097090)

**TNFR2 expression predicts the responses to** **immune checkpoint inhibitor treatments**

Ping Liao^1†^, Mengmeng Jiang^1†^, Md Sahidul Islam^1^, Yiru Wang^1^, Xin Chen^1,2,3,4*^

^†^Ping Liao and Mengmeng Jiang contributed equally to this work.

***Corresponding author:** Dr. Xin Chen, State Key Laboratory of Quality Research in Chinese Medicine, Institute of Chinese Medical Sciences, University of Macau, Macau SAR 999078, China. email: [xchen@um.edu.mo](mailto:xchen@um.edu.mo).

**Supplementary information**

**Figure 1. The expression profile of TNFR2 across varies of cancer types.**

**Figure 2. The expression profile of TNF in CD4 and CD8 T cells.**

**Supplementary Figure 1. The expression profile of TNFR2 across varies of cancer types.** TNFR2 expression in CD8 T cell metaclusters (**A**), and CD4 T cell metaclusters (**B**) across 17 cancer types were shown. (AML, acute myeloid leukemia; BCC, basal cell carcinoma; BCL, B-cell lymphoma; CHOL, cholangiocarcinoma; CRC, colorectal cancer; ESCA, esophageal cancer; FTC, fallopian tube carcinoma; HNSCC, head and neck squamous cell carcinoma; MM, multiple myeloma; NPC, nasopharyngeal carcinoma; OV, ovarian cancer; PACA, pancreatic cancer; RC, renal carcinoma; SCC, squamous cell carcinoma; STAD, stomach adenocarcinoma; THCA, thyroid carcinoma; UCEC, uterine corpus endometrial carcinoma.)


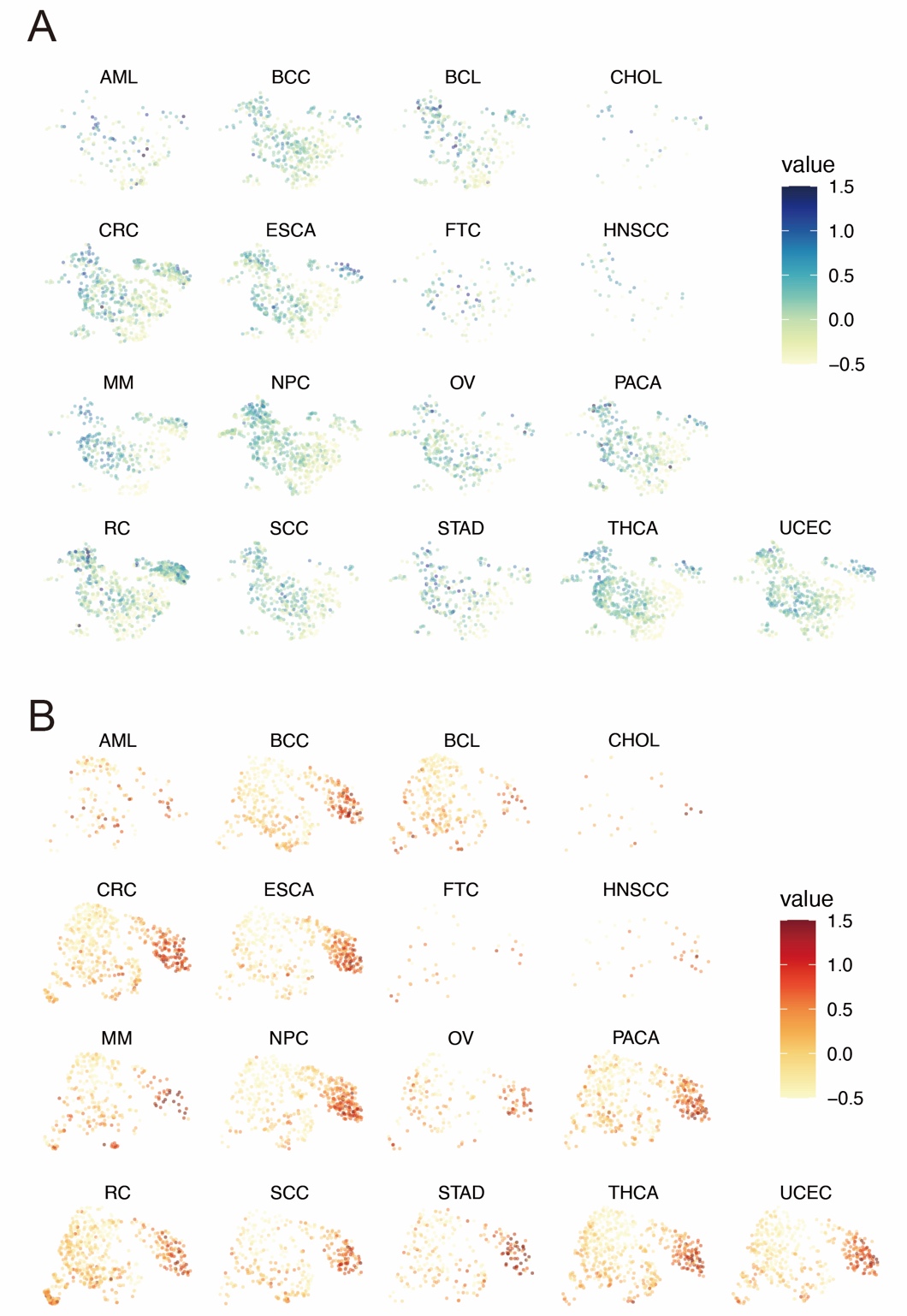


**Supplementary** **Figure 2. The expression profile of TNF in CD4 and CD8 T cells.** TNF expression in CD4 T cell metaclusters (**A**), and CD8 T cell metaclusters (**B**) were shown.


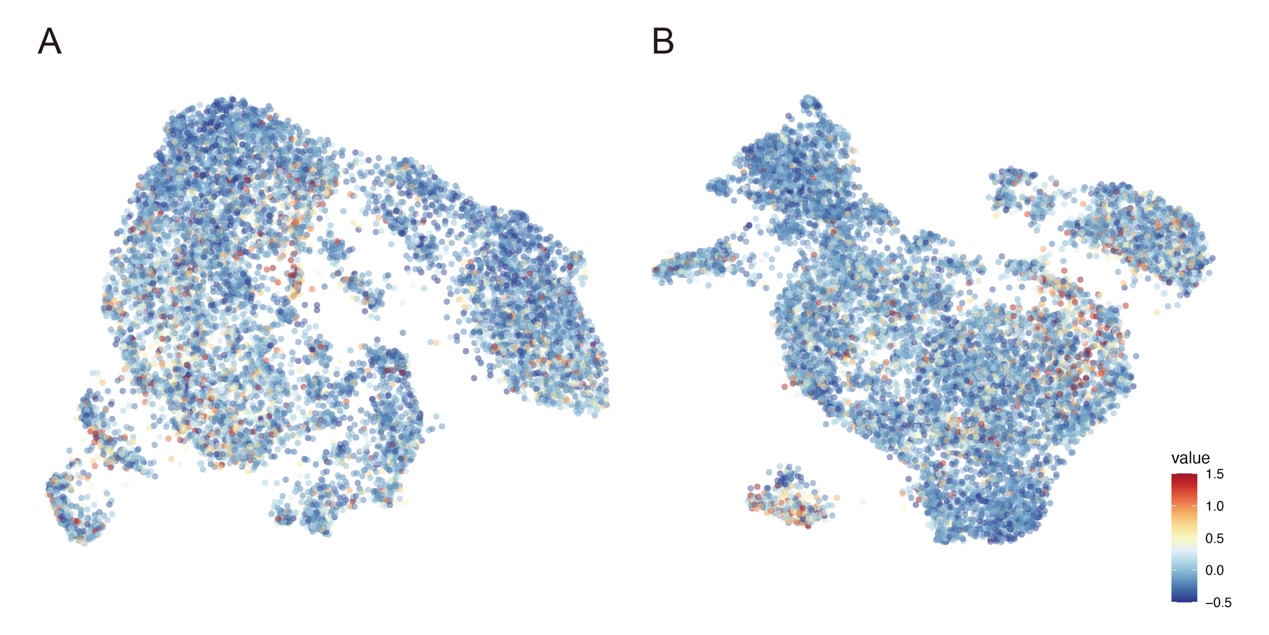

Supplement: Supplementary file 1 [file DataSheet_1.docx]
